# Mosquito control at a tertiary teaching hospital in Nigeria

**DOI:** 10.1016/j.infpip.2021.100172

**Published:** 2021-09-04

**Authors:** Akinwale M. Efunshile, Chiedozie Kingsley Ojide, Daniel Igwe, Blessing Onyia, Pikka Jokelainen, Lucy J. Robertson

**Affiliations:** a Department of Medical Microbiology, Ebonyi State University, Abakaliki, Nigeria; b Department of Medical Microbiology, Alex Ekweme Federal University Teaching Hospital, Abakaliki, Nigeria; c Laboratory of Parasitology, Department of Bacteria, Parasites & Fungi, Infectious Disease Preparedness, Statens Serum Institut, Artillerivej 5, Copenhagen S, 2300, Denmark; d Parasitology, Department of Paraclinical Science, Faculty of Veterinary Medicine, Norwegian University of Life Sciences, PO box 5003, Ås, 1432, Norway

**Keywords:** Hospital, Mosquito control, Nigeria, Patients, Questionnaire

## Abstract

**Background:**

Mosquitoes are vectors of numerous diseases, including malaria and yellow fever. Mosquito control is therefore a priority in many countries, especially in healthcare settings. Here we investigated the opinions of patients and staff regarding mosquito control at a hospital in Nigeria, and also gathered data on mosquito-control measures in this setting.

**Methods:**

We conducted a cross-sectional questionnaire study of staff and patients and an observational approach to obtain data on mosquito-control measures used at a tertiary teaching hospital in Abakaliki, Nigeria.

**Discussion:**

Both staff (N=517) and patients (N=302) reported experiencing more mosquito bites at the hospital than elsewhere. As well as contributing to discomfort, this exposure may put hospital staff and patients at risk of mosquito-borne infections. Complaints from patients about mosquitoes were reported by over 90% of staff, and over 50% of staff respondents were aware of patient discharge against medical advice due to mosquitoes. The most common control method was killing mosquitoes by hand. We observed a lack of door screens in all wards, window screens were absent or torn, and most beds did not have nets. In the children's wards none of the beds had nets.

**Conclusions:**

Current measures against mosquitoes in this hospital appeared inadequate, and healthcare staff and hospital patients may be at increased risk of mosquito-borne infections. Mosquito control in the hospital requires attention, and the needs for improvement in mosquito control in the healthcare setting more widely should be evaluated and addressed.

## Introduction

Mosquitoes are major vectors of numerous diseases, particularly, but not exclusively, in tropical regions. Diseases include Chikungunya, dengue fever, filariasis, malaria, West Nile encephalitis, yellow fever, and Zika virus infections. By transmitting diseases, mosquitoes are estimated to be responsible for causing 725,000 deaths annually [1], and considerable resources are directed towards mosquito control. Mosquito-control methods that are efficient and cost-effective include: (i) eliminating breeding places and larval habitats by drainage, oil application, or use of predators such as fish, (ii) use of exclusion barriers such as mosquito nets, particularly insecticide-treated bed nets (ITNs) and window screens, and (iii) application of insecticide sprays, especially indoor residual spraying (IRS). A modelling-based exploration found that although cost-effective interventions for malaria are available, using a suite of interventions to decrease the malaria burden may not be affordable in those countries where this is most needed [2]. Although more sophisticated methods, such as sterile-insect-technique and incompatible-insect-technique innundative release strategies [3], use of nanoparticles [4], and genetic-editing approaches [5] have also been shown to be promising, using ITN alone results in a sizeable reduction in the malaria burden, one of the most important mosquito-borne diseases [1].

According to the 2019 World Malaria Report from the World Health Organization (WHO), much of the global malaria burden occurs in Nigeria, accounting for 23% of world malaria deaths (over 88,000 in Nigeria) and 27% of malaria cases (over 61 million in Nigeria) in 2019 [6]. The objectives of Nigeria's National Malaria Strategic Plan, 2014–2020 [7], to reduce malaria burden to pre-elimination levels and reduce malaria-related mortality to zero by 2020, have not been attained. Furthermore, Nigeria is a high-risk country for another important mosquito-borne disease, yellow fever, with many areas below herd-immunity thresholds [8]. During 2019, Nigeria suffered successive yellow fever outbreaks [9], and further outbreaks were reported in 2020 [10] and 2021 (https://www.ncdc.gov.ng/diseases/sitreps/?cat=10&name=An%20update%20of%20Yellow%20Fever%20outbreak%20in%20Nigeria).

Use of ITNs, particularly long-lasting insecticidal nets (LLIN), has been a cornerstone of both WHO and Nigerian policies for curbing malaria, and millions of LLIN have been distributed (e.g., 7.3 million distributed in two Nigerian states during July and August 2019; https://www.afro.who.int/news/malaria-over-73-million-long-lasting-insecticide-treated-nets-distributed-taraba-and-kaduna). Regular use of bed nets is now common, with over 80% of respondents in one survey reporting using bed nets most or every night [11]. In Abakaliki, Nigeria, a survey of caregivers showed that over 80% had at least one ITN in their homes, although not necessarily used nightly [12].

Given that health services care for the diseased, and thus vulnerable, it is especially important that adequate control against malaria and other mosquito-borne diseases and infections is implemented in healthcare settings, including hospitals. Furthermore, nuisance biting and irritation from the whine of mosquitoes at night may hinder sleep, which is relevant for patient recovery. Positioned at the frontline in tackling mosquito-borne diseases, hospitals are places where good knowledge about the role of mosquitoes in disease transmission would be expected and appropriate strategies implemented. However, this does not always seem to be the case. For example, in an area of Thailand endemic for *Aedes* mosquitoes (vector for dengue and Zika viruses), a survey found that over 50% of hospitals had water-holding containers containing *Aedes* mosquito larvae [13]. Not only may patients and hospital staff be bitten by mosquitoes in hospital environments, but patients hospitalized due to mosquito-borne diseases may serve as sources of infectious agents that the mosquitoes may transmit further.

Anecdotal evidence has indicated that mosquito control measures may be insufficient in some Nigerian healthcare settings. We therefore decided to investigate this at a tertiary teaching hospital, by conducting a questionnaire study of staff and patients and by gathering data on mosquito-control measures used in the hospital.

## Methods

### Ethical considerations

Approval for this study was obtained from the ethical review board of the Alex Ekweme Federal University Teaching Hospital, Abakaliki, Nigeria. Participation was voluntary and anonymous; personal contact information was not collected. By returning the questionnaire, participants gave consent for their answers to be used for research purposes. All questions were entirely voluntary to answer. One exclusion criterion, evaluated on a case-by-case basis, was critical illness or distress; all other patients, with the exception of those in the neonatal ward and babies, were invited to participate. For paediatric patients, the information was collected directly from the children by trained assistants in the presence of parents or guardians, who also counted bites on the forearm wrote in the answers provided by the respondents. The publication only contains aggregated results and no personal data.

### Study design and setting

The cross-sectional study was conducted at Alex Ekweme Federal University Teaching Hospital Abakaliki (AEFUTHA), Abakaliki, South-West of Nigeria, during April to June 2019, that is, during the mosquito season (the rainy/wet season in Ebonyi State runs from April to October). The study included questionnaires for staff and patients as well as observations at ward- and patient-bed-levels. AEFUTHA has a catchment of around 780,000 residents of Abakaliki city, and, according to its homepage (https://www.aefutha.gov.ng), a 720 patient-bed capacity and an outpatient load of around 8,000 monthly.

### Sample size

Using an online survey sample size calculator (http://fluidsurveys.com/university/survey-sample-size-calculator/), with 95% confidence interval and a margin of error of 5%, we calculated that a sample of 278–385 respondents per target group (staff, patients) would be sufficient to represent the two groups. We did not adjust for clustering, e.g., by ward. We used convenience sampling: all staff and patients were invited to participate during the study period.

### Survey instrument

Structured questionnaires were designed, pre-tested on staff at Department of Medical Microbiology, edited for clarity, printed, and then distributed to the staff and patients. Both questionnaires (Appendices A and B) collected demographic information and self-reported history of malaria episodes (as assessed by symptoms and recovery after treatment, rather than laboratory diagnosis) during the previous 6 months. Hospital staff were asked about their responsibilities and hours of duty, and patients about their reasons for hospital admission, as well as duration of admission. All participants were asked whether they were bitten more by mosquitoes at home or in the hospital, and which mosquito control measures they used in the hospital. Moreover, they were also asked to report the number of mosquito bites on the left forearm at the time of questionnaire completion; where possible these numbers were confirmed by visual checking by the research assistants collecting the information. Determining when the bites had occurred was not attempted.

In addition, hospital staff were asked whether they considered mosquito bites to be a problem in the hospital, whether they received complaints from patients about mosquitoes, and whether they were aware of patients who had requested discharge against medical advice (DAMA) due to mosquitoes. All questionnaires were administered by trained assistants who also were able to assist respondents with low literacy skills or who did not understand English, by translation into the language in which the respondent felt comfortable; the assistants recorded the information in such cases.

Information on the availability and adequacy of door screens, window screens and bed nets, in the wards was obtained by visiting each ward once during the study period to count the nets and to record whether they were torn. In addition, patients were asked whether the hospital had provided the nets or whether they had brought a net themselves (and, thus, would take it with them when they were discharged).

### Statistical analyses

The data were entered in Excel for descriptive statistics, and we used open-source epidemiological calculators: 95% confidence intervals (CI, Mid-P exact) were calculated and contingency table analyses (2-tailed *P* values, Mid-P exact) were performed using https://www.openepi.com. Where appropriate, *P* values are reported in results; significance was considered when *P* ≤ 0.05.

## Results

### Description of respondents

Completed questionnaires were obtained from 517 hospital staff and 302 hospitalized patients; taken the 750-bed capacity, the patient sample represented around 40% of the patients at the time of survey. All questionnaires that were administered were returned, but not all questions were answered in each questionnaire.

Among hospital staff respondents, the majority (62%) were female. Most (63%) were aged between 26-39 years, and 25% were between 40 and 54 years. Most staff respondents were nurses (41%), while house officers and residents made up 10% and 14%, respectively. Most staff worked in the wards (23%), with substantial proportions working in paediatrics (17%), obstetrics and gynaecology (14%), laboratory medicine (12%), and surgery (11%). Hours of duty were usually shift or on-call (33%), covering all periods of the day, including night work, while 25% and 21% reported morning/afternoon work or night work, respectively. Taking shift work into account, over 50% of staff worked during night hours. Among the 517 hospital staff completing the questionnaire, three did not provide information on malaria episodes in the previous six months, but of the 514 who responded, 64 (13%) had reportedly not had any episodes of malaria in this period, 107 (21%) reported one episode, 223 reported two to three episodes (43%), and 120 (23%) reported more than three episodes.

Among the 302 patient respondents, the majority (73%) were female. Most (38%) were aged between 26–39 years, and all age groups were represented: 31% were 25 years or younger, 17% were 40–54 years, and 14% were older than 54 years. Most respondents (26%) had been admitted to the female medical ward, with 11% admitted to obstetrics and gynaecology, 11% to paediatrics, and 10% to maternity. Many patients (31%) had been in hospital for between four and seven days when the questionnaire was administered, but substantial proportions had been there for two to three days (28%), one to two weeks (17%), or more than two weeks (24%). Among patient respondents, 30 did not provide information on malaria episodes in the previous six months, but of the 272 who responded, 44 (16%) had reportedly not had any episodes of malaria in this period, 58 (21%) reported one episode, 96 reported two to three episodes (35%), and 74 (27%) reported more than three episodes.

### More mosquito bites at the hospital than at home or elsewhere

A significantly greater proportion of both hospital staff and patients (*P*<0.0001 for both) reported experiencing more mosquito bites at the hospital than at home or elsewhere: 72% of staff (372 out of 517) and 86% of patients (260 out of 302) reported more mosquito bites at the hospital than at home or elsewhere (Table 1). However, 16% of staff and 6% of patients reported no difference between home and hospital regarding mosquito bites. Patients were significantly more likely than staff to select “more bites at hospital”, and significantly less likely to select “more bites at home” or “no difference between home and hospital” (*P*<0.0001).

**Table I:** Staff and patient responses to the question of where they experienced most mosquito bites

	Staff (N=517) n (%; 95% CI)	Patients (N=302) n (%; 95% CI)	Total (N=819) N (%; 95% CI)
More bites at hospital	372 (72.0; 67.9–75.7)	260 (86.1; 81.8–89.7)	632 (77.2; 74.2–80.0)
More bites at home	52 (10.1; 7.7–12.9)	15 (5.0; 2.9–7.9)	67 (8.2; 6.5–10.2)
No difference between hospital and home for bites	81 (15.7; 12.7–19.0)	19 (6.3; 3.9–9.5)	100 (12.2; 10.1–14.6)
More bites at another location	7 (1.4; 0.6–2.7)	5 (1.7; 0.6–3.6)	12 (1.5; 0.8–2.5)
Don't know/not answered^a^	5 (1.0; 0.4–2.1)	3 (1.0; 0.3–2.7)	8 (1.0; 0.5–1.8)

95% CI, 95% confidence interval (Mid-P Exact).

a One staff respondent and one patient respondent did not answer this question.

Among the staff, 54% knew of cases of DAMA due to mosquito biting, and 94% had received complaints from patients about mosquitoes. Moreover, 94% of staff respondents considered mosquito bites at the hospital a problem.

### Mosquito bite counts on left forearm

Among hospital staff, 133 (26%) reported that they did not have mosquito bites on their left forearm at the time of investigation, 175 (34%) had fewer than five bites, and 209 (40%) between five and nine bites. None reported ten or more bites. Among patients, 14 (5%) reported no mosquito bites on their left forearm at the time of investigation, 37 (12%) had fewer than five bites, 69 (23%) between five and nine bites, and 182 (60%) at least ten. Thus, a significantly smaller proportion of patients than staff reported no bites (*P*<0.0001). In addition, high numbers of bites were reported by a significantly higher proportion of patients than staff (*P*<0.0001).

Although numbers of patients per ward were limited (between two and 79, median 18), it is worth noting that in the female medical ward, the male orthopaedic ward, the children's emergency room, obstetrics and gynaecology, and the sickle cell ward, all patients in the study reported at least some mosquito bites. Table 2 shows the number of mosquito bites according to duration of stay in the hospital. Among the 192 patients who had been in hospital for seven days maximum, 98 (51%) had at least 10 bites, whereas of the 110 patients who had been admitted for over a week (eight days minimum), a significantly higher percentage (84; 76%; *P*<0.0001) reported having at least 10 bites.

**Table II:** Duration of admission and number of mosquito bites on left forearm among patients

Duration of hospital stay in days (number of patients)		Number of mosquito bites reported on left forearm No. patients (%) by duration of hospital stay^a^			
		0 bites	<5 bites	6-9 bites	At least 10 bites
1	(n = 15)	1 (6.7)	2 (13.3)	6 (40.0)	6 (40.0)
2–3	(n = 83)	2 (2.4)	14 (16.8)	24 (28.9)	43 (51.8)
4–7	(n = 94)	4 (4.3)	13 (13.8)	28 (29.8)	49 (52.1)
8–14	(n = 39)	0 (0)	4 (10.3)	5 (12.2)	30 (76.9)
>14	(n = 71)	7 (9.9)	4 (5.6)	6 (8.5)	54 (76.0)
Total	(N = 302) (CI)	14 (4.6) (2.7–7.5)	37 (12.3) (8.9–16.3)	69 (22.9) (18.4–27.8)	182 (60.3) (54.7–65.7)

a 95% confidence interval (CI), Mid-P Exact, presented for the overall (Total) proportions: n (%) (95% CI).

### Presence of door screens, window screens, and bed nets in the hospital

None of the wards had mosquito screens in the doorways. Among 14 wards, 10 had window screens, but these were torn and evaluated as inadequate (Table 3). Bed nets were lacking for 91% of occupied patient beds, including all beds in the wards treating children (children's emergency room, paediatric ward, and postnatal ward) (Table 3). Among those occupied patient beds that had mosquito nets, most (57%) were torn (Table 3). Only in the male orthopaedic ward were there more patient beds with bed nets than without (65% with bed nets); however, most bed nets (66%) were torn. Just over 50% of bed nets had been provided by the hospital.

**Table III:** Use of mosquito netting in the wards

Ward	Door screens	Window screens	Bed nets			
			N occupied beds	Absent n (%)	Present but torn n (%)	Present and intact n (%)
Male medical	Absent	Absent	22	17 (77.3)	2 (9.1)	3 (13.6)
Male surgical	Absent	Absent	9	8 (88.9)	0 (0.0)	1 (11.1)
Female medical	Absent	Absent	79	78 (98.7)	1 (1.3)	0 (0.0)
Female surgical	Absent	Absent	11	10 (90.9)	0 (0.0)	1 (10.1)
Male orthopaedic	Absent	Present but torn	23	8 (34.8)	10 (43.5)	5 (21.7)
Psychiatric	Absent	Present but torn	7	7 (100.0)	0 (0.0)	0 (0.0)
Antenatal	Absent	Present but torn	14	13 (92.9)	1 (7.1)	0 (0.0)
Children's emergency room	Absent	Present but torn	10	10 (100.0)	0 (0.0)	0 (0.0)
Postnatal	Absent	Present but torn	24	24 (100.0)	0 (0.0)	0 (0.0)
Obstetrics & Gynaecology	Absent	Present but torn	32	31 (96.9)	0 (0.0)	1 (3.1)
Paediatric	Absent	Present but torn	32	32 (100.0)	0 (0.0)	0 (0.0)
Maternity	Absent	Present but torn	29	28 (96.6)	0 (0.0)	1 (3.4)
Sickle Cell	Absent	Present but torn	2	0 (0)	2 (100)	0 (0)
Burns & plastic surgery	Absent	Present but torn	8	8 (100)	0 (0)	0 (0)
Total	Absent in all wards	Absent in 4 of 14 wards	302	274 (90.7)	16 (5.3)	12 (4.0)

### Mosquito control measures reportedly used

**Figure 1:**
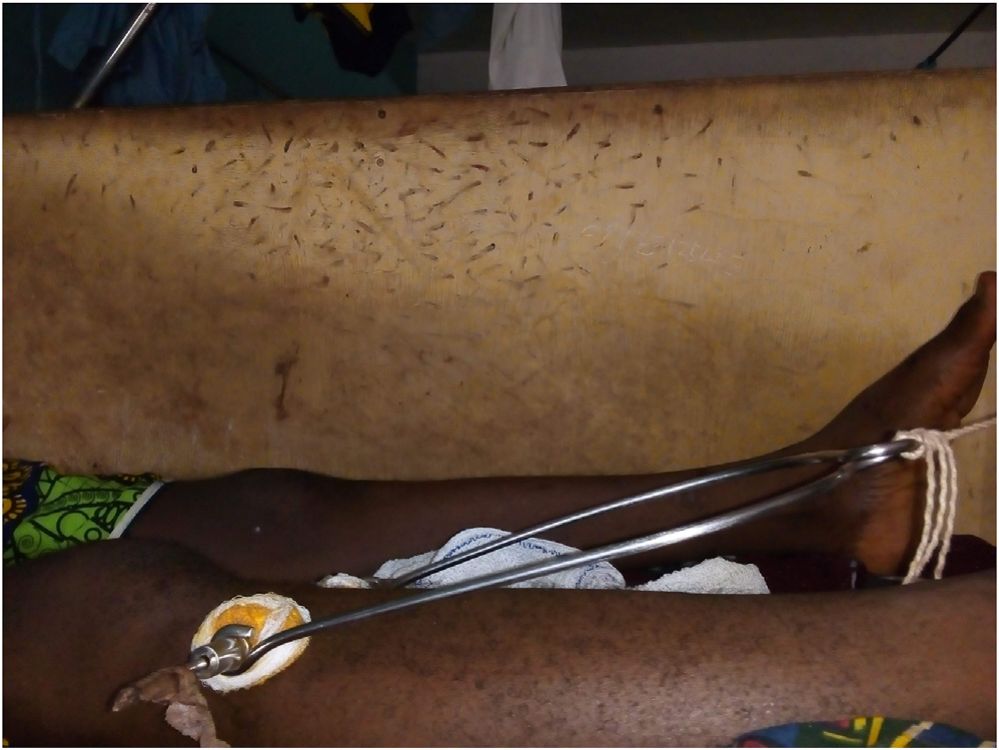
Photograph of bedside wall in the orthopaedic ward of a hospital in Nigeria showing numerous apparent marks of mosquitoes killed by patients. These can be seen as small dark smears towards the top of the wall behind the patient's leg.

Various control measures were reported by both hospital staff and patients to control mosquitoes and mosquito bites at the hospital. The most common method for both groups was killing mosquitoes by hand: this was the only method used by 217 (42%) of staff and 156 (52%) of patients. Observations of the walls beside the patient beds (Figure 1) illustrated the use of this method.

In addition to killing mosquitoes by hand, staff reported using wearing long-sleeved clothes (18%), and using repellent cream (8%) and insect sprays (7%). Among patients, the proportions using these methods were 5%, 2%, and 3%, respectively.

## Discussion

The main finding of our study, that both staff and patients experience elevated mosquito bites while at this major tertiary teaching hospital, indicates a need for improving mosquito control measures. As well as contributing to discomfort, this exposure to mosquito bites may put hospital staff and patients at risk of mosquito-borne infections. Given that patients in the hospital may be admitted due to such infections, they will provide a source of infection for staff and other patients. A more complex study from Kenya in which several hospitals were included in a study of mosquito diversity, abundance, and viruses within the mosquitoes noted that patients who are carriers at such hospitals may start local transmission chains through capable mosquito vectors [14]. A previous investigation on maintenance management in 46 public hospitals in southwestern Nigeria [15] concluded that the staff strength of maintenance departments in hospitals was frequently inadequate; inexperience and low motivation were also indicated. We did not investigate these aspects in our study, but such a situation might contribute to the inadequate mosquito control measures identified in our study. In the study from Kenya [14], insecticide and topical repellent were the main control measures within the hospital, along with grass cutting and clearing bushes; the use of insecticide-treated nets supply by the Ministry of Health was also reported and observed.

Of particular concern was that in wards treating children, who are particularly susceptible to mosquito-borne diseases, nets were not observed at any of the beds. The squashed remains of mosquitoes over the walls of the wards (Figure 1) provided a graphic statement about the lack of adequate mosquito control and illustrated also that many of the mosquitoes killed had already had a blood meal.

Despite both staff and patients reporting that they were bitten more in the hospital than at home or elsewhere, it is interesting that this was reported significantly more by patients than staff. Moreover, patients were more likely to have a high number of bites. We speculate that this difference may reflect that staff may be better able to protect themselves or, because they are moving around, they are less often bitten; patients are more likely to be less mobile and less able to physically avoid being bitten. Indeed, feverish patients may attract mosquitoes and other symptoms may suppress mosquito avoidance activities. This may result in increased mosquito bites [16]. In addition, staff may be more aware of the conditions at their workplace and thus better prepared, and staff areas may have equipment such as fans or air conditioning, which may reduce mosquito biting.

The self-reported occurrence of malarial episodes did not differ between staff and patients. However, as the information referred to the previous six months, these data should not be associated with being at the hospital but rather to reflect similarity in overall risk for malaria. Although a single bite from an infective mosquito may result in development of malaria, not all infective mosquitoes with salivary gland sporozoites are equally infectious; those with higher numbers of salivary-gland sporozoites are more likely to cause infection [17]. Thus, greater numbers of mosquito bites need not necessarily correlate exactly with malaria development. Furthermore, host factors, as well as vector factors, are relevant for transmission [18].

Although the species of mosquitoes in the location were not identified in our study, and an entomological survey could be key to developing more targeted control strategies, a previous survey from Abakaliki indicated that *Anopheles gambiae* occurs most abundantly (35%), followed by *Anopheles funestus* (*brucei*) (28%), and *Culex quinquefasciatus* (24%) [19]. The two *Anopheles* species occur widely in Nigeria and are considered important vectors of malaria in the country [20]. Although *C. quinquefasciatus* does not transmit malaria, it is an important vector of other pathogens such as *Wucheria bancrofti* (causing lymphatic filariasis) and Zika virus, both relevant pathogens in Nigeria. Thus, a substantial proportion of mosquitoes at the study location are likely to be capable of transmitting malaria and other diseases.

One weakness of our study is that, due to limited resources, only one hospital was included. Although other hospitals within the region may be in a similar situation, our results should not be extrapolated to all hospitals in Nigeria. The experiences from this study can help in planning future studies, optimally multicentre studies. A further weakness of the study was that where or when bites had occurred could not be definitively determined. Indeed, it should be noted that recall bias about where they were bitten may have affected the results, although the large number of participants and many-fold differences in our study would suggest that the main conclusions are unlikely to be affected. Our data would have been strengthened by approaches such as human landing catches (HLC) at both residential sites and within the hospital environment; however, due to the practical and financial limitations of our study it was not possible to conduct such investigations. It should be noted however, that HLC data are also prone to collector bias, and the method is labour-intensive and exposes the collectors to mosquito-borne infections [21]. Another limitation is that some bites noted on the left forearm of respondents may not have been from mosquitoes, but from other dipterans, resulting in higher counts than correct. On the other hand, bites are sometimes not easily visible and whether they are visible depends on individual reactivity; thus, the observed and reported numbers may also be underestimates. Furthermore, given that mosquitoes (including *Anopheles* species and *C. quinquefasciatus*) are largely night-biting and both staff and patients are more likely to be awake at the hospital, they may be more likely to notice both mosquitoes and being bitten there than at home.

## Conclusion

Mosquito control is important in all public buildings, including hospitals, and is of particular importance in places where mosquitoes are likely to be vectors of diseases such as malaria. The results of our cross-sectional study indicate a need to strengthen mosquito control in the hospital where the study was conducted, most importantly to reduce the risk of transmission of mosquito-borne infections, but also to lessen discomfort to both patients and staff.

## Ethical considerations

Approval for this study was obtained from the ethical review board of the Alex Ekweme Federal University Teaching Hospital, Abakaliki, Nigeria. Participation was voluntary and anonymous. The publication only contains aggregated results and no personal data.

## CRediT authorship contribution statement

**Akinwale M. Efunshile**: Administration, Conceptualization, Methodology, Supervision, Data curation, Formal analysis, Validation, Writing – review and editing. **Chiedozie Kingsley Ojide**: Supervision, Validation, Writing – review and editing. **Daniel Igwe**: Investigation, Data curation, Writing – review and editing. **Blessing Onyia**^:^ Investigation, Data curation, Writing – review and editing. **Pikka Jokelainen:** Methodology, Supervision, Data curation, Formal analysis, Validation, Writing – review and editing. **Lucy J. Robertson:** Administration, Methodology, Supervision, Data curation, Formal analysis, Validation, Visualization, Writing – original draft, Writing – review and editing.

## Appendix A Supplementary data

Multimedia component 1 Questionnaire used for hospital staff. Multimedia component 1

Multimedia component 2 Questionnaire used for hospital patients. Multimedia component 2
