# Supplementary material for: Mosquito control at a tertiary teaching hospital in Nigeria
Source: Infect Prev Pract. 2021 Sep 4;3(4):100172. doi: 10.1016/j.infpip.2021.100172 (PMC8473772; doi:10.1016/j.infpip.2021.100172)
Supplement: Multimedia component 1 — Questionnaire used for hospital staff. [file mmc1.docx]

Study on hospital-acquired mosquito bites

QUESTIONNAIRE FOR HEALTHCARE WORKERS

Dear participants, we are investigating mosquito bites among healthcare workers and patients in this hospital. Information provided is confidential and for research purpose only, and will not affect you or your work in any way.

Thank you for answering this questionnaire. Please tick the appropriate boxes for the questions below.

Contact person:-Dr. Efunshile Akinwale - 09084164194

1. **Personal and professional**
   1. **Gender**

Male Female

- 1. **Age group**

25 years or under 26 years to 39 years 40 years to 54 years 55 years or older

- 1. **Position in the hospital**

Consultant Resident doctor Medical officer Consultant Nurse

Lab scientist Other (please specify)-…………………………………………………………………………

1. **Department**
2. **In which department do you currently work**

Surgery Medicine O & G Paediatrics

Lab medicine Other (please specify)…………………………………………………………………………..

1. **Usual working hours in the hospital (you can choose more than one option)**

Morning (08:00 until noon) Afternoon (noon until 19.00)

Night (19:00 until 07:00) Other (please specify)…………………………………

1. **Mosquito bites and malaria and you**
2. **Where do you experience more mosquito bites?**

At home At hospital Elsewhere Same at home and hospital

Don’t know

1. **Do you consider mosquito bites at this hospital to be a problem?**

Yes, definitely Somewhat/slightly Not really No, not at all

I don’t know

1. **If bitten at work in the hospital, are there are any working periods particularly affected?**

No difference Don’t know Morning (08:00 until noon) Afternoon (noon until 19.00) Night (19:00 until 07:00) Other (please specify)…………………

1. **Do mosquitoes influence your ability to perform your duties?**

Yes, a lot Somewhat/slightly No Don’t know

1. **How do you control against mosquito bites while on duty? (You can choose more than one option)**

No control Repellant cream Insecticide spray Mosquito coil

Treated bed net Long-sleeved clothes Other (please specify)………………………………….

1. **How many malaria episodes did you experience in the last 6 months?**

None One Two or three More than three

1. **Please write here the number of mosquito bites currently visible on your left forearm (from elbow to fingers, including hand and fingers)……………………….**
2. **Mosquito bites patients**
3. **Have patients ever complained to you about mosquito bites at this hospital?**

Yes No Don’t know

1. **Have patients in your care ever requested for “discharge against medical advice” (DAMA) because of mosquitoes?**

Yes No Not applicable
