# Supplementary material for: Mosquito control at a tertiary teaching hospital in Nigeria
Source: Infect Prev Pract. 2021 Sep 4;3(4):100172. doi: 10.1016/j.infpip.2021.100172 (PMC8473772; doi:10.1016/j.infpip.2021.100172)
Supplement: Multimedia component 2 — Questionnaire used for hospital patients. [file mmc2.docx]

Study on hospital-acquired mosquito bites

QUESTIONNAIRE FOR PATIENTS

Dear participants, we are investigating the mosquito bites among healthcare workers and patients in this hospital. Information provided is confidential and for research purpose only, and will not affect you or your care in the hospital in any way.

Thank you for answering this questionnaire. Please tick the appropriate boxes for the questions below.

Contact person:-Dr Efunshile Akinwale-09084164194

1. **Personal**
   - 1. **Gender**

Male Female

- - 1. **Age group**

25 years or under 26 years to 39 years 40 years to 54 years 55 years or older

- - 1. **Education (highest level reached)**

primary secondary university or college

**Your current hospital admission**

1. **Name of ward…………………………………………………………….**
2. **How long have you been in hospital:**

1 day 2-3 days 4 days to 1 week 1-2 weeks more than 2 weeks

1. **Mosquito control in this hospital**

**a) Where do you experience more mosquito bites?**

At home At this hospital Elsewhere Same at home and hospital

Don’t know

1. **Have you been bitten by mosquitoes during your current hospital admission?**

Yes, a lot Yes, somewhat Not much Not at all Don’t know

1. **Is an insecticide treated bed net (ITN) available for your use during this admission?**

Yes No Don’t know

1. **If answer to b) is yes (ITN available), do you always use it?**

Yes No Don’t know Not relevant (no ITN available)

If “No”, please give reason:…………………………………………………………………………………………

1. **How do you control against mosquito bites here in the hospital?** (You can tick more than one option)

No control Repellant cream Insecticide spray Mosquito coil

Treated bednet Long-sleeved clothes Other (please specify)………………………………

1. **Have mosquitoes disturbed your sleep during your staying in this hospital?**

Yes, definitely Somewhat/slightly Not really No, not at all

I don’t know Not relevant (have not stayed overnight)

1. **Should you need to be admitted to hospital again, will mosquito bites at this hospital influence your hospital choice?**

Yes No I don’t know

**h) Please write here the number of mosquito bites currently visible on your left forearm (from elbow to fingers, including hand and fingers)……………………….**

1. **Malaria**
2. **How many malaria episodes have you experienced in the last 6 months?**

None One Two or three More than three

1. **Have you suffered a malaria attack during this hospital admission?**

Yes No I don’t know

**To be completed by Research Assistant, regarding the ward where patient has been admitted:**

1. **Is there a mosquito net at the door?**

No Yes, but torn or inadequate Yes, adequate and in reasonable condition

1. **Is there mosquito netting at the windows?**

No Yes, but torn or inadequate Yes, adequate and in reasonable condition

1. **Is there a mosquito net hanging at the bed?**

No Yes, but torn or inadequate Yes, adequate and in reasonable condition

Thank you!
